# The comparison of DBS and RNS for adult drug-resistant epilepsy: a systematic review and meta-analysis

**DOI:** 10.3389/fnhum.2024.1429223

**Published:** 2024-06-19

**Authors:** Qinghua Li, Yongzhi Shan, Penghu Wei, Guoguang Zhao

**Affiliations:** ^1^Department of Neurosurgery, Xuanwu Hospital, Capital Medical University, Beijing, China; ^2^Clinical Research Center for Epilepsy Capital Medical University, Beijing, China; ^3^Beijing Municipal Geriatric Medical Research Center, Beijing, China

**Keywords:** epilepsy, deep brain stimulation, responsive neurostimulation, neuromodulation, meta analysis

## Abstract

**Objective:**

Neuromodulation has been proven to be a promising alternative treatment for adult patients with drug-resistant epilepsy (DRE). Deep brain stimulation (DBS) and responsive neurostimulation (RNS) were approved by many countries for the treatment of DRE. However, there is a lack of systematic studies illustrating the differences between them. This meta-analysis is performed to assess the efficacy and clinical characteristics of DBS and RNS in adult patients with DRE.

**Methods:**

PubMed, Web of Science, and Embase were retrieved to obtain related studies including adult DRE patients who accepted DBS or RNS. The clinical characteristics of these patients were compiled for the following statistical analysis.

**Results:**

A total of 55 studies (32 of DBS and 23 of RNS) involving 1,568 adult patients with DRE were included in this meta-analysis. There was no significant difference in seizure reduction and responder rate between DBS and RNS for DRE. The seizure reduction of DBS and RNS were 56% (95% CI 50–62%, *p* > 0.05) and 61% (95% CI 54–68%, *p* > 0.05). The responder rate of DBS and RNS were 67% (95% CI 58–76%, *p* > 0.05) and 71% (95% CI 64–78%, *p* > 0.05). Different targets of DBS did not show significant effect on seizure reduction (*p* > 0.05). Patients with DRE who accepted DBS were younger than those of RNS (32.9 years old vs. 37.8 years old, *p* < 0.01). The mean follow-up time was 47.3 months for DBS and 39.5 months for RNS (*p* > 0.05).

**Conclusion:**

Both DBS and RNS are beneficial and alternative therapies for adult DRE patients who are not eligible to accept resection surgery. Further and larger studies are needed to clarify the characteristics of different targets and provide tailored treatment for patients with DRE.

## 1 Introduction

Epilepsy is one of the most common serious neurological diseases, affecting about 70 million people of the world population of all ages and ethnicities (Devinsky et al., 2018; Thijs et al., 2019; Rincon et al., 2021). It is now considered as an abnormality of the brain networks and caused a serious burden on society. In 2017, the International League Against Epilepsy (ILAE) updated the classification and terminology of epilepsy, which may contribute to a more comprehensive understanding of epilepsies (Scheffer et al., 2017). Despite over twenty antiseizure drugs (ASDs) for the treatment of epilepsy, about one-third of epilepsy patients are pharmacotherapy-resistant (Devinsky et al., 2018; Thijs et al., 2019). Patients with drug-resistant epilepsy (DRE) have higher risks of unexpected injuries, neurological impairment, decreased quality of life, and even death. It is of urgent need to develop more effective therapies for DRE patients. Seizure onset zone (SOZ) resection surgery and laser ablation are options for patients who have definite SOZ. However, for those DRE patients with multi-foci seizures or not eligible for resective surgeries, neuromodulation provides them with new prospects (Rincon et al., 2021; Ryvlin et al., 2021).

Three neurostimulation methods including vagus nerve stimulation (VNS), deep brain stimulation of the anterior nucleus of the thalamus (ANT-DBS), and responsive neurostimulation (RNS) had been approved by the United States Food and Drug Administration (FDA) in the treatment of DRE (Ryvlin et al., 2021; Touma et al., 2022). As the first neuromodulation device approved for treating DRE, VNS was used worldwide. The efficacy and safety of VNS were demonstrated by numerous randomized controlled trials and retrospective studies (Touma et al., 2022). Though the mechanisms of ANT-DBS in epilepsy are still unclear, it was proposed that it might partly correlate with the role of the thalamus within the Papez circuit and relation with temporal and frontal cortical regions (Yan et al., 2022). The SANTE trial has demonstrated the effect of ANT-DBS for DRE (Fisher et al., 2010; Salanova et al., 2021). Over the past decades, electrodes were placed in different nuclei and relays to identify the efficacy of DBS in treating DRE. The convincing findings of the SANTE trial led to the recognition and acknowledgment of the effectiveness of ANT-DBS (Fisher et al., 2010; Salanova et al., 2021). This comprehensive study, conducted at multiple medical centers, was carried out by Fisher et al. and involved 110 patients suffering from either focal or focal to generalized seizures (Fisher et al., 2010). Until now, ANT is the only approved stimulation target of DBS by the FDA for the treatment of DRE in adult DRE patients (Zhu et al., 2021; Alcala-Zermeno et al., 2022). Velasco et al. performed the initial study exploring the potential benefits of stimulating the CM in the treatment of patients with DRE (Velasco et al., 2006). The use of CM-DBS appears to hold great promise in the management of absence and generalized seizures, particularly in individuals diagnosed with Lennox Gastaut syndrome (LGS) (Dalic et al., 2022). Interestingly, approximately 80% of patients with LGS exhibited a positive response to CM-DBS (Torres Diaz et al., 2021; Alcala-Zermeno et al., 2022). The ANT, the centromedian nucleus of the thalamus (CM), and the hippocampus (HIP) ranked among the most frequently employed targets, while the majority of the studied targets is the ANT (Hodaie et al., 2002; Lee et al., 2012; Oh et al., 2012; Bondallaz et al., 2013; Cukiert et al., 2014; Salanova et al., 2015; Lim et al., 2016; Son et al., 2016; Kim et al., 2017; Herrman et al., 2019; Schaper et al., 2019; Guo et al., 2020; Alcala-Zermeno et al., 2021; Cukiert et al., 2021; Parisi et al., 2021; Passamonti et al., 2021; Salanova et al., 2021; Thuberg et al., 2021; Torres Diaz et al., 2021; Wang et al., 2021; Zhu et al., 2021; Alcala-Zermeno et al., 2022; Dalic et al., 2022; Olaciregui Dague et al., 2023).

The RNS System can record long-term and real-time electroencephalography or electrocorticography data by implanted electrodes. It was originally designed as a closed-loop brain stimuSlation system to deliver short bursts of stimulations depending on brain electrical activities (Barbaro et al., 2019; Jarosiewicz and Morrell, 2021). After the safety verification of 65 patients in an open-label feasibility trial, a comprehensive RCT study conducted by Heck et al. demonstrated that RNS (NeuroPace) significantly reduced TLE and focal epileptic seizures (Heck et al., 2014). The safety and efficacy of RNS in treating DRE were also demonstrated by the following studies (Anderson et al., 2008; Heck et al., 2014; Chen et al., 2017; Geller et al., 2017; Jobst et al., 2017; Kerolus et al., 2017; Razavi et al., 2020; Tran et al., 2020; Zawar et al., 2021; Alcala-Zermeno et al., 2022; Brown et al., 2022; Chen et al., 2022).

VNS is the only FDA approved modality in the USA for pediatric DRE, while ANT-DBS and RNS are approved for adult patients (Thijs et al., 2019; Ryvlin et al., 2021). DBS and RNS are less commonly implanted in DRE patients than VNS, given the shorter history of clinical use in epilepsy. Until now, there is rare systematic reviews concentrating on the comparison of DBS and RNS for DRE. Besides, there is no consensus on the choice of clinical use in patients with DRE (Ryvlin et al., 2021; Cross et al., 2022). In recent years, emerging evidence has suggested the reliability of DBS and RNS. We conducted this meta-analysis to clarify the comparisons of DBS and RNS in the treatment of DRE and provide evidence for future studies.

## 2 Materials and methods

### 2.1 Data source and search strategy

This systematic review was performed following Preferred Reporting Items for Systematic Reviews and Meta-Analyses (PRISMA) guidelines (Liberati et al., 2009). The databases of PubMed, Web of Science, and Embase were systematically searched for relevant studies published between January 2000 and October 2023, specifically in the English language. The search strategy for DBS included the terms “Deep brain stimulation” or “DBS” AND “epilepsy” or “seizure.” For RNS, the search terms used were “Responsive neurostimulation” or “RNS” AND “epilepsy” or “seizure.” Additionally, the references within relevant studies and reviews were also retrieved and identified to ensure comprehensive coverage and avoid any omissions.

### 2.2 Inclusion and exclusion criteria

Studies that met the inclusion criteria were enrolled for the next step. The inclusion criteria were: (1) RCT, retrospective studies, prospective studies, or observational studies illustrating seizure frequency before and after DBS or RNS; (2) patients must be at least 18 years old with identified DRE; (3) studies with the description of seizure reduction and responder rate; (4) published in English. The exclusion criteria were: (1) number of patients was less than 5; (2) letters, reviews, editorials, and abstracts.

### 2.3 Data extraction and quality assessment

The following information was extracted from the included studies: the author, publication year, neuromodulation device, number of DRE patients, age at surgery, seizure duration, follow-up, seizure reduction (SR), responder rate (seizure reduction rate greater than 50%, RR), and adverse event. SR was calculated by the seizure frequency of the last follow-up compared with the baseline. The full text of the included articles was screened by two reviewers independently (LQH and WPH). Clinical data were extracted from the articles. Variables were calculated from the original data when information was not clearly stated. The disagreement between reviewers was solved by consulting the third author (ZGG). The Newcastle-Ottawa Scale (NOS) (Stang, 2010) was employed to assess the quality of the included studies, which comprises three main components: selection, comparability, and outcome. Each study with a score of NOS over 7 indicating high quality.

### 2.4 Statistical analysis

All statistical analyses were performed using STATA 14 SE (StataCorp, College Station, TX, USA). The primary outcomes were SR and RR. The heterogeneity between included studies was calculated with the Q test and I^2^ statistic. A random-effects model was used when there was a high heterogeneity Q test (*p* < 0.05) or I^2^ > 50%. The mean SR and standard deviation (SD) of several studies were estimated according to the median and interquartile range (IQR). A sensitivity analysis was also conducted to detect the effect of a single study on final results. Publication bias was examined by funnel plot.

## 3 Results

### 3.1 Search results

**Figure 1:**
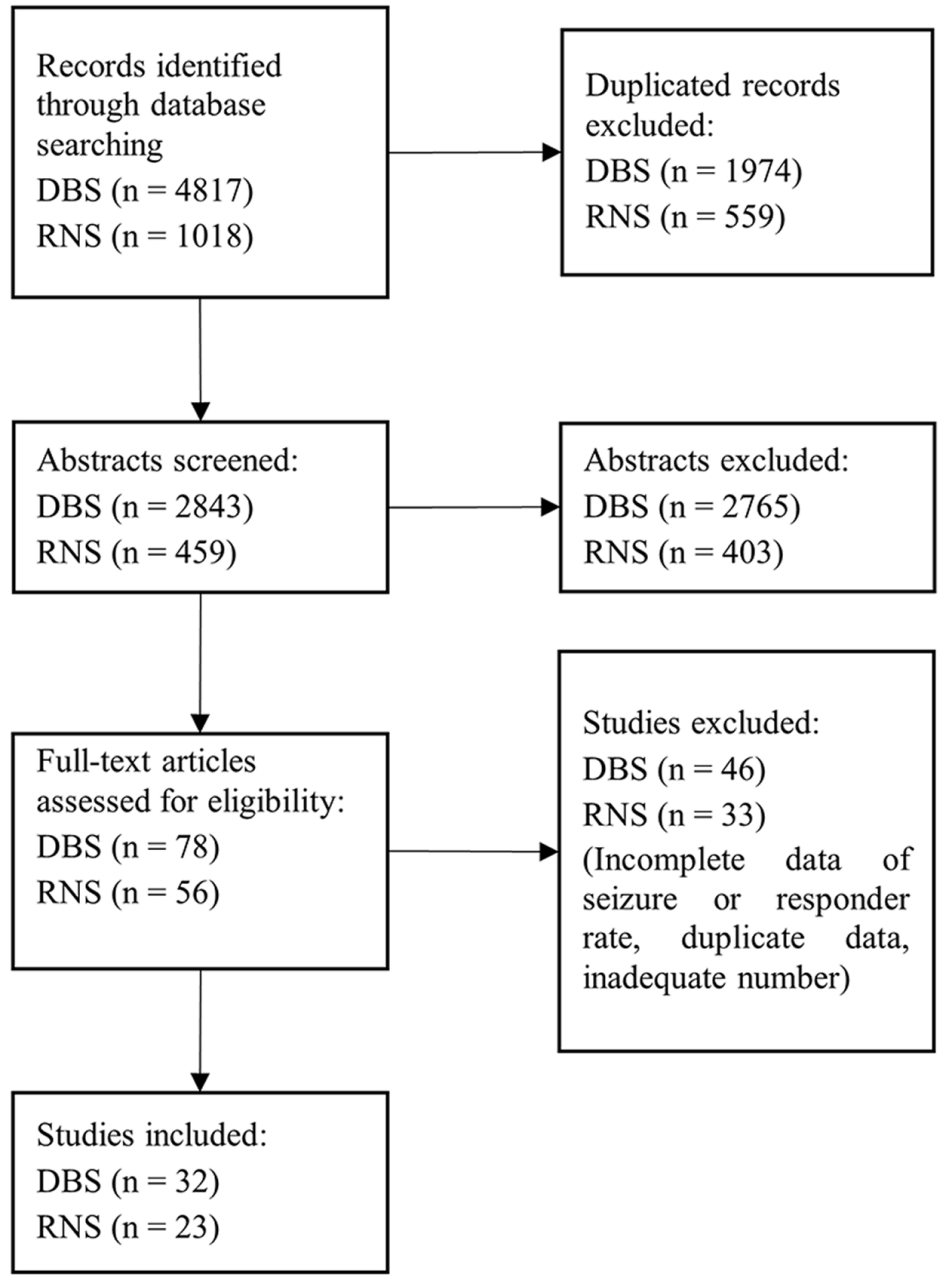
PRISMA flowchart of the study selection.

The process of identifying relevant studies based on PRISMA is shown in Figure 1. The retrieving process yielded 5,835 studies. 2,992 studies were excluded after screening the abstracts. The remaining 134 studies were reviewed for full text. During the exclusion process, 23 studies were excluded due to the inadequate number of participants, 32 studies due to incomplete data of SR or RR, 12 studies were reviews, and 11 studies were performed in children. Finally, 55 articles were included for further statistical analysis (32 for DBS and 23 for RNS), as shown in Tables 1, 2. Given the long-term open-label trials of the RNS system were published separately for different periods, studies of Jobst et al., Geller et al., and Nune were included. The study published by Nair et al. (2020) was not included on account of incomplete data on many patients losing contact.

**Table 1:** Basic characteristics of DBS studies included.

Author	Year	Target	Study type	*N*	Age (mean ± SD)	Duration (years)	FU^a^ (months)	SR^b^	RR^c^	AE^d^
Hodaie	2002	ANT^e^	Case	5	30.4 ± 10.5	28.4 ± 10.6	14.9 ± 4.4	0.55 ± 0.27	0.60	NA
Velasco	2007	CM^f^	Retrospective	8	31 ± 8.3	19.9 ± 10.2	NA	0.87 ± 0.18	1	0
Fisher	2010	ANT	RCT^g^	110	36.1 ± 11.2	22.3 ± 13.3	36 ± 14.4	0.56	0.67	NA
Oh	2012	ANT	Retrospective	9	33.4 ± 11	29 ± 16.4	34.6 ± 16.6	0.58 ± 0.17	0.78	NA
Vonck	2013	HIP^h^	Prospective	11	NA^i^	NA	102	0.66 ± 0.39	0.27	0.27
Lee	2012	ANT	Retrospective	15	30.5 ± 12.2	17.0 ± 11.1	39.0 ± 14.33	0.71 ± 0.32	0.87	0
Bondallaz	2013	HIP	Retrospective	8	34.1 ± 2.8	NA	43.5	0.67 ± 0.37	0.75	NA
Cukiert	2014	HIP	Prospective	9	37.2	NA	30.1	0.69 ± 0.45	0.78	NA
Salanova	2015	ANT	RCT	75	36.1	22.3	NA	0.49	0.68	NA
Son	2016	CM	Retrospective	14	29 ± 10	NA	18.2 ± 5.6	0.68 ± 0.22	0.79	0
Lim	2016	HIP	Case	5	35.2 ± 14.6	19.4 ± 9.4	38.4 ± 5.4	0.45 ± 0.22	0.60	0
Cukiert	2017	HIP	RCT	16	38.4 ± 13.6	NA	6	0.37 ± 0.56	0.88	NA
Kim	2017	ANT	Retrospective	29	29 ± 16.5	19.3 ± 9.0	72	0.739	0.76	0.31
Herrman	2018	ANT	RCT	18	NA	24	12	0.23 ± 0.27	0.22	0.17
Schaper	2019	ANT	Prospectively	10	41.9 ± 9.6	27.9 ± 11.8	12	0.43 ± 0.55	0.50	NA
Cukiert	2021	HIP	Prospective	25	39	NA	57	0.68 ± 0.25	0.72	NA
Wang	2021	HIP	Retrospective	7	33.4 ± 11.1	13.1 ± 10.4	48 ± 36	0.78 ± 0.27	0.86	0
Thuberg	2021	ANT	Prospective	14	37.4 ± 10.1	NA	25.1 ± 8.8	0.57 ± 0.30	0.50	NA
Parisi	2021	ANT	Retrospective	33	32 ± 15.2	18.2 ± 14.2	25.5 ± 26	0.55 ± 0.32	0.67	0.55
Vázquez	2021	Subiculum	Observational	6	28.8 ± 8.6	NA	24	0.49 ± 0.42	0.5	0.33
Zhu	2021	ANT	Retrospective	18	28.9 ± 12	15.4 ± 9.4	12	0.65 ± 0.30	0.72	0
Diaz	2021	CM	Retrospective	10	30.8 ± 5.9	NA	83.8 ± 41.9	0.51 ± 0.30	0.80	NA
Dalic	2022	CM	RCT	20	25 ± 6.3	21.7 ± 7.4	86.5	0.54 ± 0.31	0.40	0.35
Passamonti	2021	ANT/STN/CM	Retrospective	6	39.2 ± 3.5	NA	84	0.18 ± 0.29	0.33	NA
Salanova	2021	ANT	RCT	73	37.1 ± 11.8	22.5 ± 13.9	120	0.75	0.74	0.13
Guo	2020	ANT	Retrospective	19	34.8 ± 9.9	19.3 ± 9.0	23.8 ± 7.5	0.64 ± 0.28	0.79	NA
Miron	2022	ANT	Prospective	11	31.2 ± 8.6	21.4 ± 7.8	28.7 ± 13.2	0.47 ± 0.31	0.72	0.36
Tong	2022	ANT	Retrospective	11	23.9 ± 10.6	11.3 ± 7.9	12	0.51 ± 0.61	0.55	NA
Alvarado	2022	PHC^j^	RCT	6	29.3 ± 12.5	17.3 ± 3.9	12	0.41 ± 0.14	0.50	0
Poulen	2022	ANT	Retrospective	9	35.8 ± 10.6	23.8 ± 8.2	47.6	0.68	0.88	0
Zermeno	2022	ANT/CM	Retrospective	57	26.3	17	24.3	0.56 ± 0.32	0.56	NA
Dague	2023	ANT	Retrospective	11	35.5 ± 8.6	21 ± 7.5	51.5	0.59 ± 0.32	0.55	NA

a FU represent the follow-up time.

b SR represent seizure reduction.

c RR represent responder rate.

d AE represent adverse event.

e ANT represent anterior nucleus of the thalamus.

f CM represent centromedian nucleus of the thalamus.

g RCT represent randomized controlled study.

h HIP represent hippocampus.

i represent not available.

j PHC represent perirhinal cortex.

**Table 2:** Basic characteristics of RNS studies included.

Author	Year	Study type	*N*	Age (mean ± SD)	Epilepsy duration (years)	Follow up (months)	Seizure reduction	Responder rate	Adverse event
Anderson	2008	Prospective	5	34 ± 10.9	NA^a^	23.2 ± 1.3	0.51	0.80	0
Heck	2014	RCT^b^	191	34.9 ± 11.5	20.0 ± 11.2	24	0.53	0.55	0.03
Chen	2017	Retrospective	7	24	NA	6	0.55 ± 0.33	0.71	0.14
Geller	2017	Prospective	111	37.3 ± 11.3	19.8 ± 12.7	73.2 ± 7.6	0.67 ± 0.46	0.65	0.12
Kerolus	2017	Prospective	8	44.6 ± 12.1	NA	9.6 ± 7.2	0.74 ± 0.16	1	NA
Jobst	2017	RCT	126	30.4 ± 10.1	19.5 ± 10.2	73.2	0.58 ± 0.63	0.55	0.07
Young	2018	Retrospective	9	34.4	NA	NA	0.4	0.75	NA
Chan	2019	Retrospective	8	44.8	15.6	14.4	0.69 ± 0.24	0.63	NA
Nune	2019	Retrospective	8	45.5 ± 10.4	15.1 ± 6	NA	0.85 ± 0.03	0.88	NA
Sisterson	2020	Retrospective	12	35.6 ± 11.9	18.5 ± 10.4	21.5 ± 10.4	0.67	0.42	NA
Wang	2020	Retrospective	12	39.2 ± 12.1	12.8 ± 9.4	43.6 ± 39.1	0.58 ± 0.39	0.67	0.13
Nunna	2020	Retrospective	10	44.6 ± 7.1	21.6	50.4 ± 11.4	0.44 ± 0.34	0.40	0.10
Razavi	2020	Retrospective	150	39	20	27.6	0.74 ± 0.34	0.84	0.11
Tran	2020	Retrospective	10	36	NA	NA	0.81 ± 0.09	1	0.2
Karas	2020	Retrospective	10	40	15	7.5	0.26 ± 0.55	0.38	0
Burdette	2020	Retrospective	7	33.4 ± 9.5	NA	16.8	0.8 ± 0.29	0.86	0.22
Ma	2020	Retrospective	30	NA	14.2 ± 10.3	21 ± 11.6	0.63 ± 0.33	0.70	0
McDermott	2021	Retrospective	5	35	NA	16.8	0.88 ± 0.17	1	NA
Zawar	2021	Retrospective	55	35.1 ± 12.9	19.5 ± 12.6	38 (6–144)	0.50 ± 0.36	0.55	NA
Brown	2022	Retrospective	64	36.0 ± 11.8	NA	27.6	0.39 ± 1.06	0.67	0.26
Zermeno	2022	Retrospective	30	39	18	19.2	0.5 ± 0.39	0.56	0
Chen	2022	Retrospective	9	40 ± 16.3	11.3 ± 6.73	31.2	0.39 ± 0.37	0.44	NA
Ho	2022	Retrospective	13	38.3 ± 10.4	25.2 ± 12.9	57.6 ± 13.2	0.71 ± 0.19	0.77	0

a NA represent not available.

b RCT represent randomized controlled study.

### 3.2 Characteristics of the included studies

Tables 1, 2 demonstrate the characteristics of included DBS and RNS studies separately. A total of 1,568 adult DRE patients were included in the final statistical analysis (678 patients of DBS and 890 patients of RNS). Selected studies were published between 2002 and 2023. The number of patients varied from 5 to 191 in these studies. In studies providing specific sex ratios, 50.6% (305/603) were female in DBS and 49.7% (442/890) in RNS studies. There is no significant gender difference between DBS and RNS studies. The results of age at surgery suggested that patients who accepted DBS were younger than those of RNS (32.9 years old vs. 37.8 years old, *p* < 0.01), while the differences of disease duration (20.4 years vs. 17.6 years, *p* > 0.05) and follow-up time (34.7 months vs. 37.5 months, *p* > 0.05) between DBS and RNS were not significant. Patients included had a wide diversity of etiologies spanning from focal epilepsies to multifocal epilepsies, Lennox–Gastaut syndrome, Tuberous sclerosis complex, and generalized epilepsies. When compared with DBS studies, there is rare RNS cases used for generalized epilepsy among the included studies. Therapeutic DBS targets, mainly determined by the institutions and experience of the medical team, varied between these studies including ANT, CM, HIP, STN, and perirhinal cortex (PHC). Stimulation targets of RNS depended on the SOZ, including the thalamic nuclei, hippocampus, neocortex, insula, and amygdala.

### 3.3 Quality assessment and publication bias

**Figure 2:**
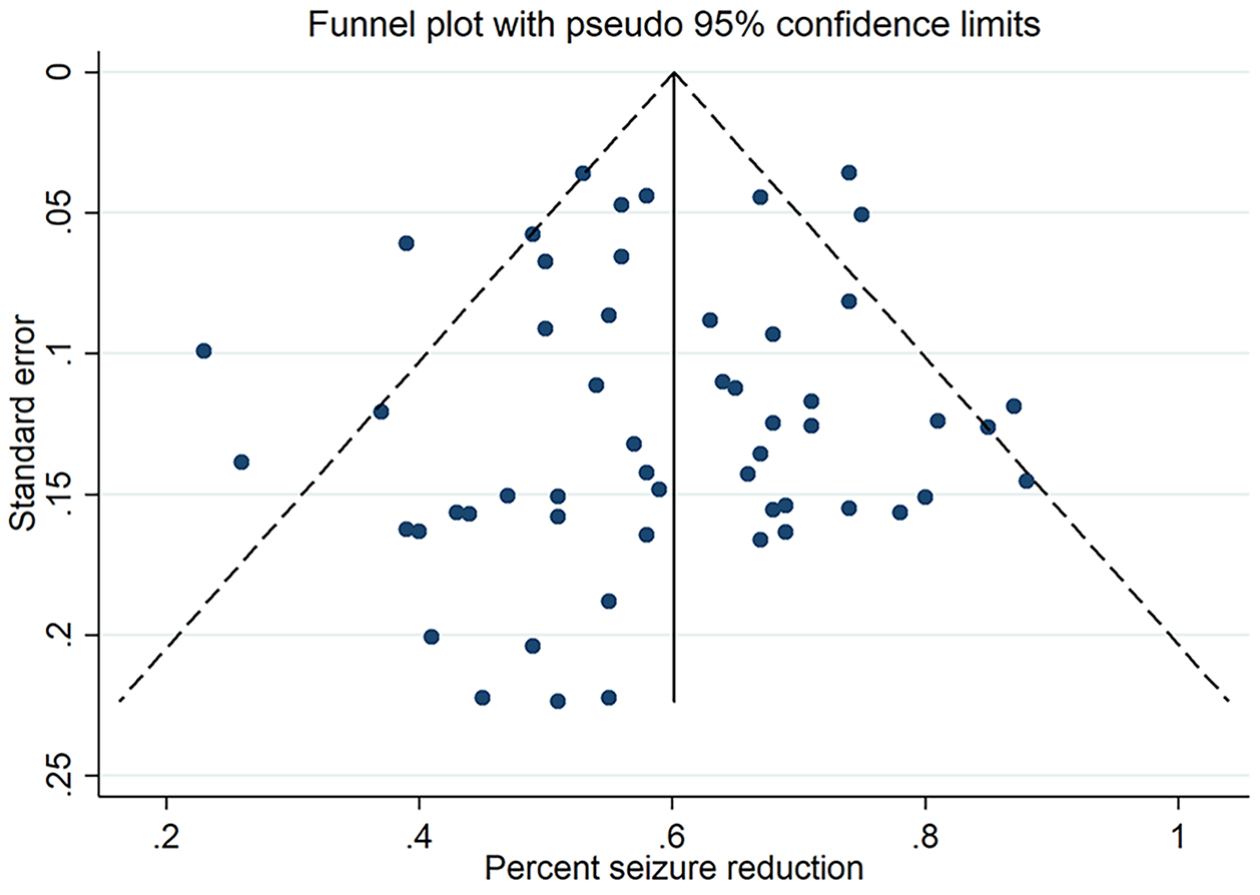
Funnel plot of publication bias of included studies.

All studies exhibited high quality according to NOS score greater than 6. Symmetric publication bias was evaluated by the funnel plots for DBS and RNS (Figure 2), and no evidence of bias was observed from it.

### 3.4 Seizure reduction and responder rate

**Figure 3:**
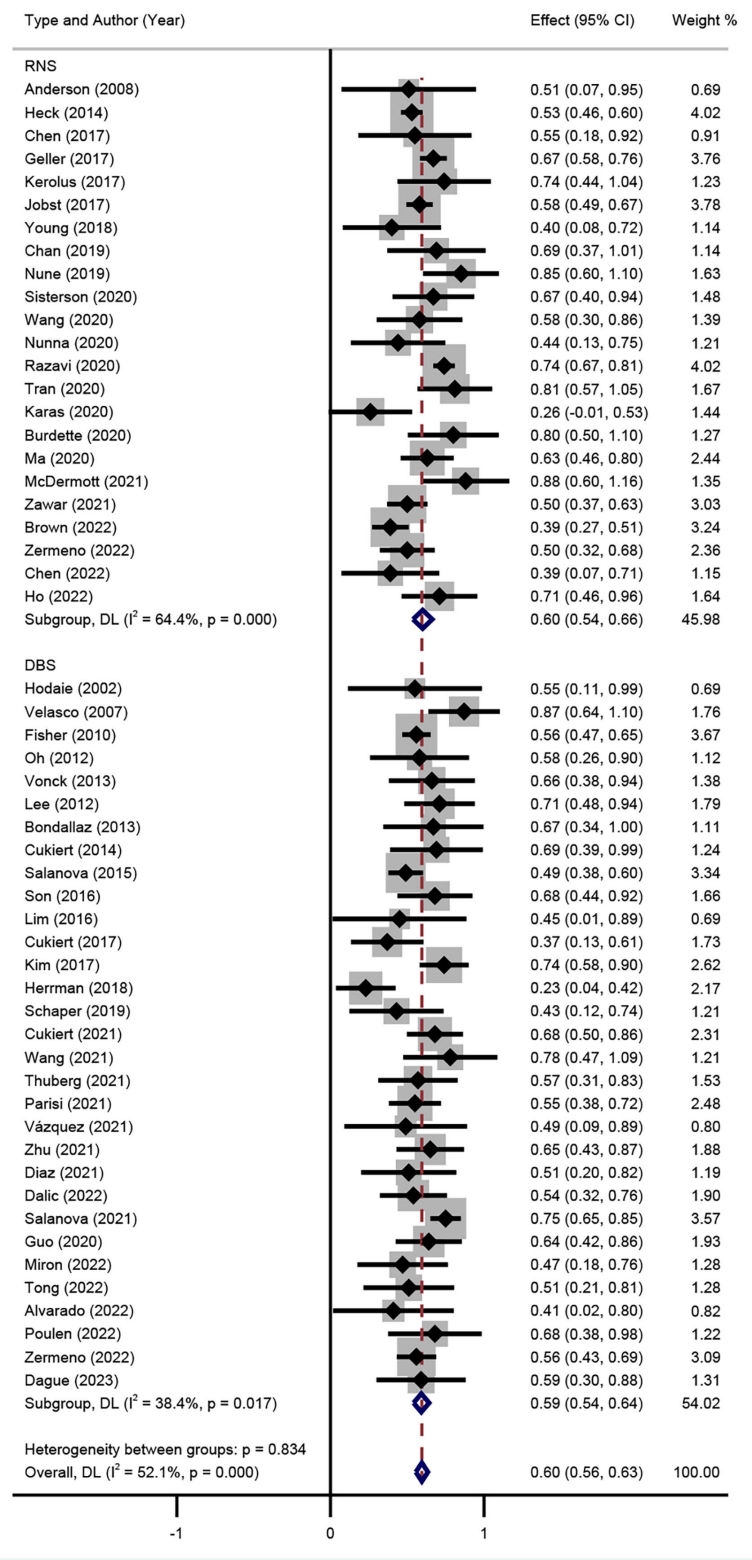
Forest plot for the comparison of the efficacy of seizure reduction between DBS and RNS.

The SR results of DBS and RNS are shown in the forest plots (Figure 3). Significant heterogeneity was detected between trials (I^2^ = 52.1%, *p* = 0.000), thus the random effect model was adopted. Patients who accepted DBS or RNS had SR of 56% (95% CI 50–62%) and 61% (95% CI 54–68%), respectively.

### 3.5 Subgroup analysis

**Figure 4:**
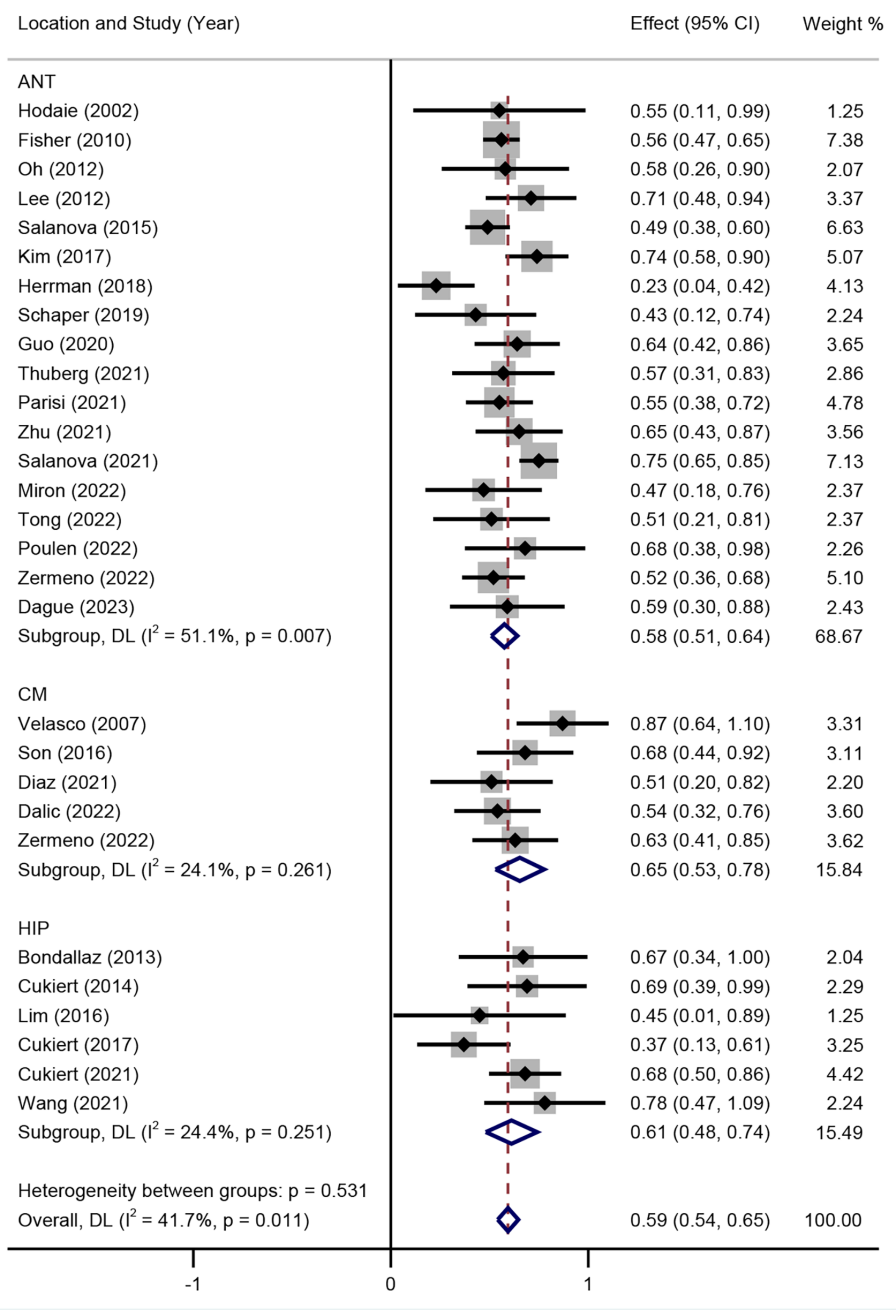
Forest plot for the efficacy of DBS in seizure reduction in different target subgroups.

Subgroup analysis of DBS targets indicated that the SR between DBS and RNS for DRE was not significant (*p* > 0.05) as shown in Figure 3, with an overall SR of 59% (95% CI 54–63%). Subgroup analysis of different stimulation targets was also performed in DBS studies (ANT, CM, and HIP). The random effect model was used (I^2^ = 41.7%, *p* = 0.011). As presented in Figure 4, different targets of DBS for DRE also revealed that there was no significant SR among ANT-DBS, CM-DBS, and HIP-DBS (*p* > 0.05). No such result was analysed given the limited number of studies clearly stated the targets of RNS.

**Figure 5:**
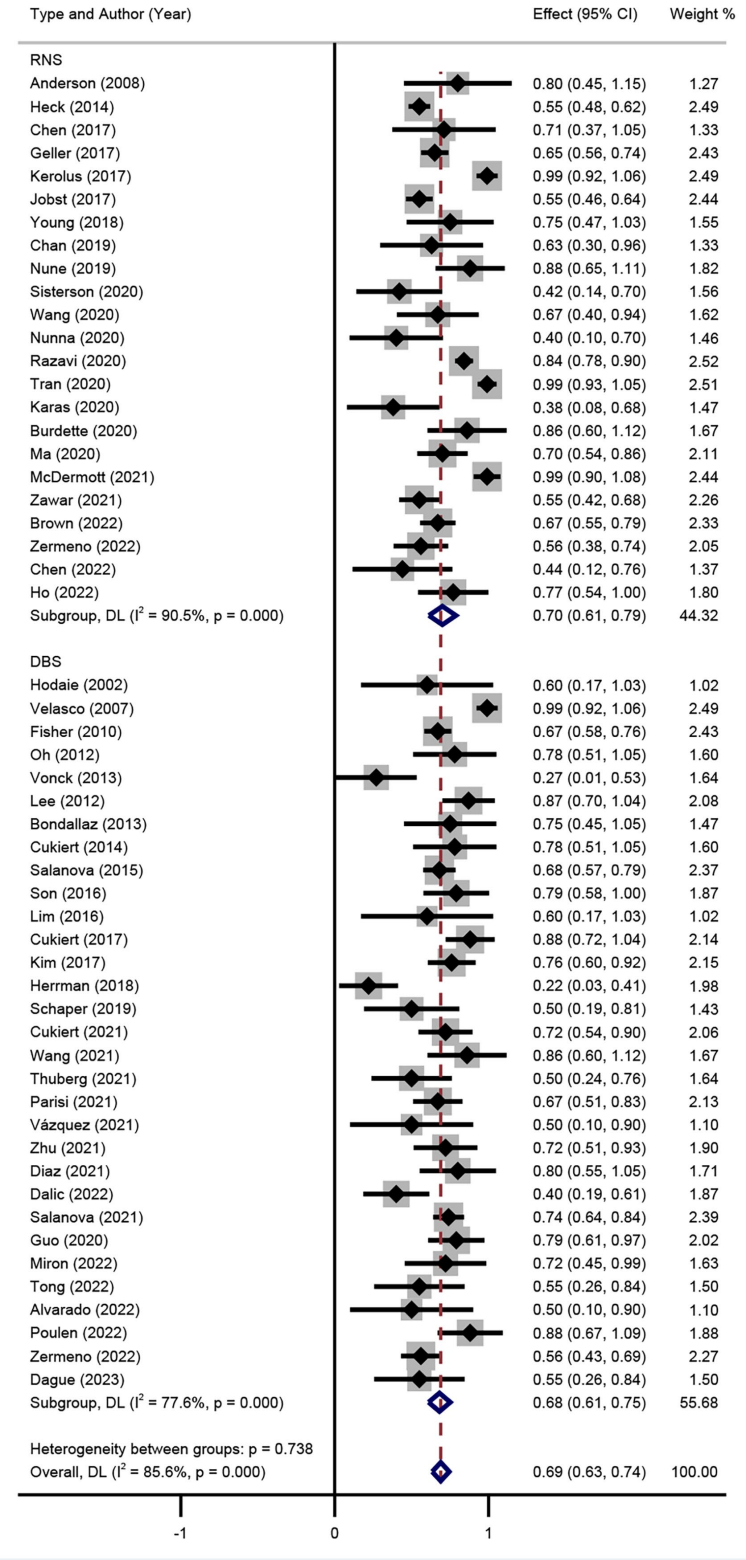
Forest plot for the comparison of the efficacy in achieving responder rate between DBS and RNS.

**Figure 6:**
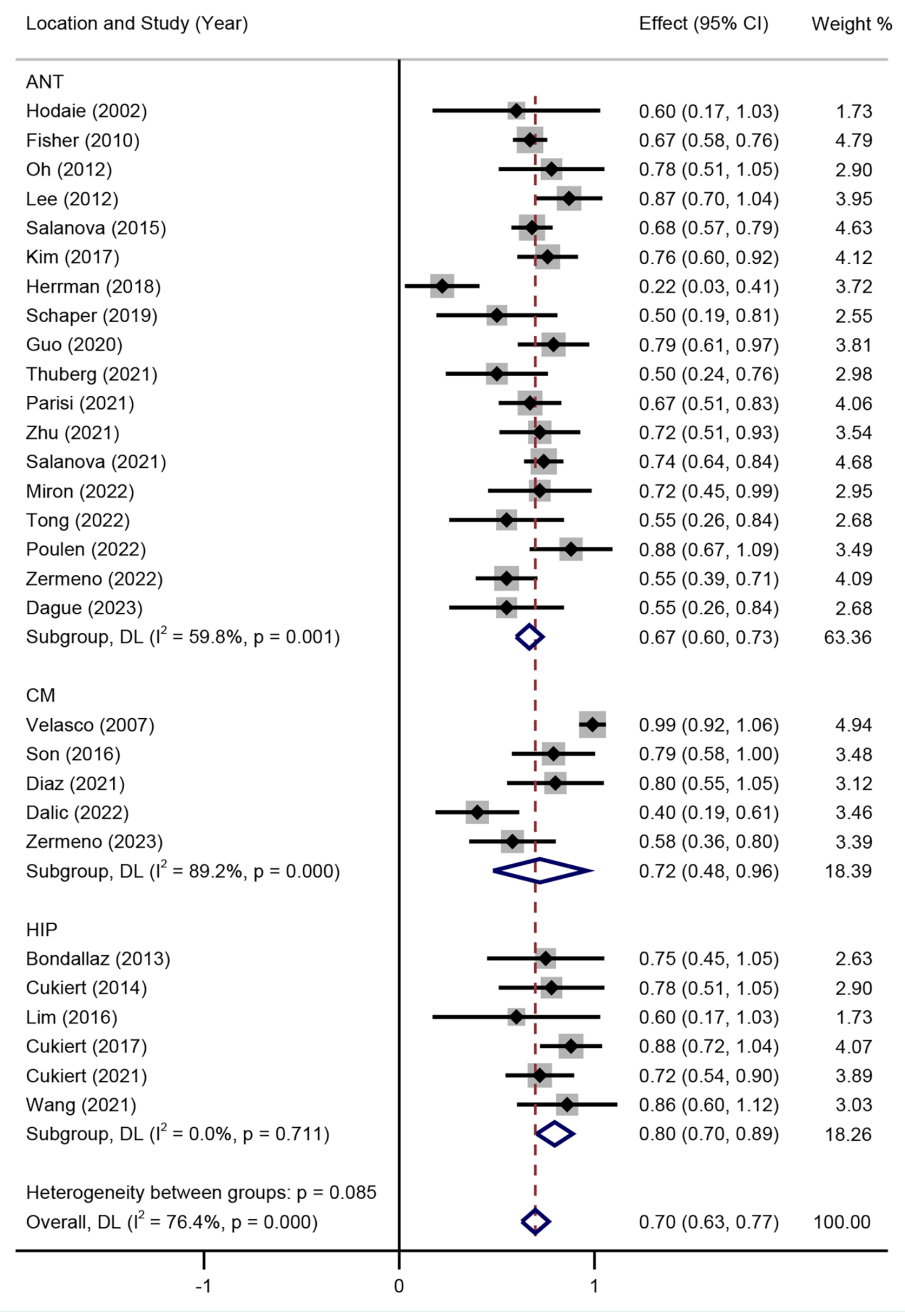
Forest plot for the efficacy of DBS in achieving responder rate in different target subgroups.

Seizure RR was also performed in the random effect model. The results of included studies showed that the RR of DBS studies was 67% (95% CI 58–76%) and 71% (95% CI 64–78%) of RNS studies (Figure 5). The result indicated that there was no significant difference between RR of DBS and RNS studies (*p* > 0.05, Figure 5). Subgroup analysis showed that there was no significant difference of RR among different DBS electrode locations (Figure 6).

### 3.6 Sensitivity analysis

Sensitivity analysis was also performed to examine the potential heterogeneity. The results indicated there was no significant contribution of each study would not change the final results.

## 4 Discussion

In the past, epilepsy was perceived as a focal brain disease. However, recent studies have gathered substantial evidence indicating that it is, in fact, a disorder that stems from an epileptogenic network (Scheffer et al., 2017; Thijs et al., 2019). With the rapid development of neuromodulation in recent years, it is becoming increasingly prevalent for DRE patients to receive neuromodulation therapy as an alternative treatment (Rincon et al., 2021; Ryvlin et al., 2021; Cross et al., 2022). Stimulation therapies present an enhanced level of control and reversibility, while maintaining a minimally-invasive approach. Within the realm of intracranial neurostimulation interventions, notable techniques such as DBS and RNS have emerged as efficacious and accessible treatment choices for reducing seizure burden in specifically chosen patients with DRE (Hodaie et al., 2002; Fisher et al., 2010; Lee et al., 2012; Oh et al., 2012; Bondallaz et al., 2013; Salanova et al., 2015; Son et al., 2016; Herrman et al., 2019; Schaper et al., 2019; Cukiert et al., 2020; Parisi et al., 2021; Rincon et al., 2021; Ryvlin et al., 2021; Torres Diaz et al., 2021; Wang et al., 2021; Zhu et al., 2021; Touma et al., 2022).

To the best of our knowledge, this is the most comprehensive study to compare the DBS and RNS for DRE. Results of this study suggested that both DBS and RNS systems are beneficial in reducing seizures (56% of DBS, 61% of RNS) and improving RR (67% of DBS, 71% of RNS), but there is no significant difference between them (*p* > 0.05). However, there is a significant difference of age at surgery between DBS and RNS (32.9 years and 37.8 years, *p* < 0.01). Numerous factors could impact the choice of patients and clinicians to select the appropriate neuromodulation system. Firstly, DBS was applied earlier in the treatment of epilepsy than RNS, which may have attracted the attention of DRE patients in the early stages. Secondly, RNS is only approved for use in the United States, while ANT-DBS is approved and widely used in North America, Europe, and a few other countries. It’s harder for DRE patients to get access to RNS than DBS. Furthermore, there were differences in the selection of patients between DBS and RNS. The RNS studies included DRE patients with localized seizures to one or two foci, while DBS could be used for multi-foci patients (Ryvlin et al., 2021). Despite lacking approval for clinical use, there is a mounting body of evidence indicating the potential effectiveness of DBS for generalized and/or multifocal epilepsies. Patients with generalized epilepsy were usually diagnosed before 25 years of age, which may also contribute to the younger age of DBS (Gesche et al., 2020). Specifically, in the RNS study, the incidence of patients who underwent invasive monitoring before implantation continued to rise in the subsequent studies, indicating either a growing preference for stereo-EEG (SEEG) prior to implantation or increased utilization of RNS in patients assessed with SEEG but deemed unsuitable for resection (Simpson et al., 2022). Besides, relatively more complex parameter adjustments of RNS pose challenges for doctors. DBS employs primary or rechargeable batteries, while RNS utilizes primary cells until now. Non-rechargeable devices are more convenient but with the shortage of more frequent battery replacements, which may cause concerns among young people. Neither duration time (19.0 years of DBS and 17.6 years of RNS, *p* > 0.05) nor the follow-up time (34.7 months of DBS and 37.5 months of RNS, *p* > 0.05) had statistically significant differences between DBS and RNS. It corresponded with the results of several studies (Salanova et al., 2015; Geller et al., 2017; Nair et al., 2020; Alcala-Zermeno et al., 2022).

### 4.1 DBS

The therapeutic effects of DBS are intricate and rely on several factors including electrical parameters, targeted regions, as well as pathological networks involved. The modulation of DBS could subsequently lead to extensive alterations within the neural networks, leading to the disruption of seizure propagation or modification of the seizure threshold (Yan et al., 2022). However, the underlying mechanism of DBS in alleviating epilepsy remains unclear. Various studies have indicated the paramount importance of the anterior thalamic region in the sustenance and spread of seizures. This can be attributed to its intricate connections with the limbic system, cerebral cortex, and caudate (Peltola et al., 2023). However, it should be noted that the results of ANT-DBS for DRE were not always satisfactory. A randomized study conducted by Hermann et al. yielded inconsistent results compared to previous studies (Herrman et al., 2019). It is believed that CM-DBS is involved in the disturbance of abnormal synchronization between the thalamus and the cortex, as well as the modulation of the arousal network. The considerable number of pathways originating from the CM region to the sensory-motor areas can potentially account for the presence of generalized seizures in these regions during functional studies (Torres Diaz et al., 2021).

Our results indicated that there was no significant difference of RR/SR between DBS and RNS. Meanwhile, subgroup analysis of different DBS targets also suggested no significant RR/SR differences between ANT-DBS, CM-DBS, and HIP-DBS. These results may be caused by several factors. Given there is no consensus on which device or target to be better, different DBS targets or RNS were chosen according to various reasons (e.g., experience of clinicians, age of patients, and seizure types). Typically, ANT-DBS seems suitable for focal seizures, while wider CM-DBS for generalized seizures (Fisher et al., 2010; Son et al., 2016; Herrman et al., 2019; Dalic et al., 2022). Besides, the follow-up time varied among the included studies. Precious studies reported that longer follow-up durations were associated with improved outcomes (Nair et al., 2020; Dalic et al., 2022). Extended follow-up durations enable researchers to conduct more comprehensive evaluations and make adjustments. The mechanisms of different DBS targets or RNS for epilepsy are still elusive. Further research is required to determine whether similar neural networks are activated by different DBS targets or RNS. Furthermore, accessibility to devices, experience of clinician, and parameter adjustment could also influence the efficacy of DBS and RNS (Herrman et al., 2019; Nair et al., 2020; Dalic et al., 2022).

Several other areas of the brain, such as the perirhinal cortex (PHC), hypothalamus, and cerebellum are less commonly used in the treatment of DRE, but have still been proven to be effective (Lee et al., 2012; Bondallaz et al., 2013; Cukiert et al., 2014; Son et al., 2016; Cukiert et al., 2020; Parisi et al., 2021). The specific position of the electrode contacts within the HIP did not show any correlation with the outcomes for either focal aware seizures (FAS) or FIAS. Moreover, there was no difference in outcomes between patients who received unilateral or bilateral HIP-DBS treatments (Cukiert et al., 2020). Promising results have been observed in reducing seizure frequencies in patients with DRE when employing different targets for DBS.

In recent years, the concept of regulating DBS based on feedback signals has generated significant interest. This approach, known as “adaptive DBS,” includes various control modes such as responsive, adaptive, and closed-loop control. The primary motivation behind the development of adaptive DBS is its potential to enhance effectiveness and mitigate adverse effects.

### 4.2 RNS

In contrast to VNS and thalamic DBS procedures, where the placement of electrodes is predetermined, RNS utilizes intracranial strip and depth electrodes that can be adjusted according to the specific location where seizures originate (Boddeti et al., 2022). RNS demonstrated a progressive and significant improvement over time. The latest 9-year follow-up report of the prospective open-label long-term treatment (LTT) clinical study (NCT00572195) was published in 2020 by Nair et al. (2020), which also observed continuous and significant SR of focal epilepsies with 75% and a responder rate of 73%. Alcala-Zermeno et al. (2022) observed no clear correlation between the specific type of neuromodulation or the type of seizure onset and improved seizure reduction or responder rates, which is consistent with our findings.

### 4.3 For pediatric patients

Although RNS and DBS have shown potential benefits in treating pediatric epilepsy, it is important to note that it has not yet received FDA approval for children. In the case of children, surgical interventions are prioritized due to the adverse effects of seizures and high doses of medications on the developing brain. DBS and RNS are considered as an option for children with DRE only when other treatment alternatives have been exhausted and seizure onsets become increasingly challenging to manage.

Subsequent outcomes in adults have exhibited improvement over time, while this has not yet been explored in the pediatric population. The greater capacity for network plasticity in children suggests the possibility of even more positive results. However, it is important to recognize certain limitations that correlate with the characteristics of children. It is worth noting that the implantation of the RNS System requires a full-thickness craniectomy, which may pose challenges in pediatric patients due to their smaller and developing skulls.

### 4.4 Adverse events of DBS and RNS

The recording and classification of adverse events of included articles varied among studies. It was mainly classified as stimulation-related, device-related, and operation-related complications. The commonly seen adverse events are similar between DBS and RNS due to their intrinsic of intracranial implantation surgery. Adverse events reported were consistent with the known risks associated with implanted medical devices, seizures, and other epilepsy treatments. Great variabilities of adverse events were observed among studies. This phenomenon may be attributed to the different sample and follow-up time.

#### 4.4.1 Hemorrhage and infection

The overall reported adverse events of hemorrhages and infections were similar between DBS and RNS (Fisher et al., 2010; Vonck et al., 2013; Geller et al., 2017; Jobst et al., 2017; Kim et al., 2017; Nair et al., 2020; Razavi et al., 2020; Tran et al., 2020; Zawar et al., 2021; Brown et al., 2022). When examining the common complications specifically, the incidence of intracranial hemorrhage ranged from 0 to 9.1% in DBS studies (Fisher et al., 2010; Vonck et al., 2013; Kim et al., 2017), while it was 0 to 10% in RNS studies (Geller et al., 2017; Jobst et al., 2017; Razavi et al., 2020; Tran et al., 2020; Zawar et al., 2021; Brown et al., 2022). The incidence of site or intracranial infections was 0 to 12.7% in DBS studies (Fisher et al., 2010; Kim et al., 2017; Parisi et al., 2021; Salanova et al., 2021; Alcala-Zermeno et al., 2022) and 0 to 12.5% in RNS studies (Jobst et al., 2017; Zawar et al., 2021; Brown et al., 2022; Chen et al., 2022; Hartnett et al., 2022). Few patients who lost their lives were related to Sudden Unexpected Death in Epilepsy (SUDEP) (Heck et al., 2014; Jobst et al., 2017). Implantation-related infection was one of the most common serious adverse events. In most cases, it necessitates the removal of the hardware.

#### 4.4.2 Cognitive impairment

During the SANTE trial, mood disorders and memory impairment were the most frequently reported adverse events related to stimulation, which was also observed in other studies (Fisher et al., 2010; Salanova et al., 2021; Wang et al., 2021; Peltola et al., 2023). At the end of the blinded period, individuals receiving active stimulation reported depression in 15% of cases and memory impairment in 13%. After 7 years of follow-up, approximately one-third of individuals experienced depression or memory impairment events. The depression symptom was also reported in several studies (Kim et al., 2017; Herrman et al., 2019; Peltola et al., 2023). Nevertheless, the alteration of cognitive function was demonstrated during DBS for epilepsy (Herrman et al., 2019; Olaciregui Dague et al., 2023). However, some researchers did not observe an obvious decline in neuropsychological function, on the contrary, verbal memory and word fluency were significantly increased after ANT-DBS (Oh et al., 2012).

It is reported that thalamic RNS have subtle negative effects on various cognitive domains, especially verbal memory. There is limited evidence to illustrate the adverse effect of RNS on mood and cognitive functions. The study reported that RNS System did not have any adverse impact on cognitive functioning, neuro-psychological function, or mood (Heck et al., 2014). Adverse events reported were consistent with the known risks associated with implanted medical devices. Roa et al. did not obtain any objective measurements or tests to evaluate cognitive and psychological effects. The participants did not suffer from negative neuropsychological effects following thalamic RNS (Roa et al., 2022). A limited number of unfavorable incidents, such as abnormal sensations (paresthesia), discomfort at the site of implantation, malfunctioning of the hardware, and the lead being positioned inaccurately, were also documented on a minor scale.

It is essential to note that the included studies lacked consistency in reporting relative complications like memory, sensory, and psychiatric disorders, rendering a more detailed statistical analysis unfeasible. This finding provides substantial evidence supporting the safety and effectiveness of DBS and RNS.

### 4.5 Limitations

This study contains several limitations. The retrospective, non-RCT, observational studies and case series enrolled in this meta-analysis have inherent limitations and are not able to provide the same level of evidence as RCTs. Various factors related to patients and procedures make it challenging to draw definitive conclusions about the outcomes of epileptic seizures. These factors include a wide range of seizure types and causes, the uncertainty surrounding patients’ own reports of their seizures, differences in research methods such as varying stimulation parameters and surgical targeting techniques, the naturally unpredictable course of the disease, and the alterations in antiepileptic medication during the follow-up period. Additionally, patient characteristics such as gender, age, and stimulation parameters were not taken into account. Meanwhile, the follow-up time varied among different studies and the SR and RR reported were not consistent, which suggests the potential existence bias of results. Furthermore, multivariable regression analysis was not performed given the limited data. Multivariable regression analysis is a powerful tool, as it allows us to control for confounding variables (e.g., age, sex, accessibility of devices, duration of epilepsy, and seizure types), assess the impact of these independent variables, identify significant predictors, quantify relationships, and adjust treatment comparisons to better understand the safety and efficacy of DBS and RNS for epilepsy.

## 5 Conclusion

In conclusion, this study demonstrates the benefits of DBS and RNS in reducing SR and improving RR for adult DRE patients. The efficacy of seizure control and safety are comparable to each other, which indicates both of them are alternative therapies for eligible DRE patients. Larger-scale and long-term follow-up RCTs are needed to further clarify the choices of epilepsy type and target locations.

## Data availability statement

The original contributions presented in the study are included in the article/supplementary material, further inquiries can be directed to the corresponding authors.

## Author contributions

QL: Writing – original draft. YS: Investigation, Writing – review & editing. PW: Writing – review & editing. GZ: Writing – review & editing.
